# Characteristics and mechanisms to control a COVID‐19 outbreak on a leukemia and stem cell transplantation unit

**DOI:** 10.1002/cam4.3612

**Published:** 2020-12-12

**Authors:** Jochen Greiner, Marlies Götz, Waltraud Malner‐Wagner, Constanze Wendt, Martin Enders, Christine Durst, Detlef Michel, S von Harsdorf, Susanne Jung

**Affiliations:** ^1^ Department of Internal Medicine Diakonie Hospital Stuttgart Stuttgart Germany; ^2^ Department of Internal Medicine III University of Ulm Ulm Germany; ^3^ Laboratory Dr. Limbach and colleagues Heidelberg Germany; ^4^ Laboratory Prof. Gisela Enders and colleagues Stuttgart Germany; ^5^ Institute of Virology University of Ulm Ulm Germany

**Keywords:** acute myeloid leukemia, COVID‐19 infection, health‐care facilities, immunocompromised individuals, management of disease control

## Abstract

Immunosuppressed patients like patients with leukemia or lymphoma, but also patients after autologous or allogeneic stem cell transplantation are at particular risk for an infection with COVID‐19. We describe a COVID‐19 outbreak on our leukemia and stem cell transplantation unit (LSCT‐Unit) originating from a patient with newly diagnosed acute myeloid leukemia. The patient was treated with intensive induction chemotherapy and we characterize the subsequent outbreak of COVID‐19 on a LSCT‐Unit. We describe the characteristics of the 36 contacts among the medical team, the results of their PCR and antibody tests and clinical aspects and features of infected employees. Of these 36 close contacts, 9 employees of the LSCT‐Unit were infected and were tested positive by PCR and/or antibody‐testing. 8/9 of them were symptomatic, 3/9 with severe, 5/9 with mild symptoms, and one person without symptoms. Due to stringent hygiene measures, the outbreak did not lead to infections of other patients despite ongoing clinical work. Moreover, we demonstrate that incubation period and clinical course of a COVID‐19 infection in an immunosuppressed patient could be unusual compared to that of immunocompetent patients. Consistent PCR and antibody testing are helpful to understand, control, and prevent outbreaks. For the safety of health‐care workers and patients alike, all employees wore FFP2 masks and were trained to adhere to several further safety guidelines. The implementation of rigorous hygiene measures is the key to controlling an outbreak and preventing infections of other patients.

## Graphical abstract

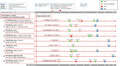

## 1 INTRODUCTION

In December 2019, the Corona Virus Disease 2019 (COVID‐19) outbreak started in China and rapidly developed into a pandemic threatening the population worldwide. COVID‐19 signifies a great risk especially for the elderly, patients with chronic diseases, and immunosuppressed patients, particularly for patients with hematological malignancies like leukemia or lymphoma and patients after autologous and allogeneic stem cell transplantation. In patients with hematological malignancies, a high mortality of COVID‐19 could be expected but at this time, there are only reports on small cohorts but no systematic clinical studies available.^1^, ^2^, ^3^, ^4^ Apart from the individual risk an infection carries for these patients, there is a high risk for all health‐care facilities that members of their staff will be infected by the virus and develop COVID‐19,^5^ thereby dangerously reducing the number of health‐care workers able to treat patients sufficiently.^6^ There also exists the additional high risk in all health‐care facilities that personnel become virus carriers and develop transmission chains between health‐care workers and patients.^7^ In particular, as asymptomatic carriers can infect other people, apparently healthy medical staff have the potential of infecting patients with severe hematological diseases during their treatment. Similarly, patients switching between outpatient and inpatient treatment could be initially asymptomatic carriers potentially turning into the source of an outbreak in the clinical units. However, due to the long incubation time, initial PCR testing is no guarantee to have noninfected patients.

In this report, we describe a COVID‐19 outbreak on our leukemia and stem cell transplantation unit (LSCT‐Unit) originating from a 59‐year‐old female patient who was newly diagnosed with acute myeloid leukemia (AML), which measures we took to control the outbreak among the employees and how we avoided further spreading of the disease.

Our patient developed fever and atypical pneumonia in aplasia. We did a swab for severe acute respiratory syndrome coronavirus 2 (SARS‐CoV‐2) detection, although at this point an infection with SARS‐CoV‐2 seemed to be rather unlikely, considering that our AML patient had been in hospital for over 10 days already. Our patient tested positive for COVID‐19. Thus, even patients who have been in hospital longer need surveillance testing for COVID‐19.^8^, ^9^

Due to the fact, that COVID‐19 was only detected after the patient had spent 2 weeks in hospital, the patient had 36 category I contacts, that is, cumulative face‐to‐face contact for at least 15 minutes, or exposure during aerosol‐forming procedures, or as part of a medical examination.

We will outline the probable path of infection in our clinic, the experiences we made, and the successful management of disease control during ongoing clinical work. The process changed during this time because of varying requirements of the local and national health‐care authorities but also due to temporary lack of assays and/or swab tubes. We describe the cluster, timeline, type and number of contacts, tests and test results and development of symptoms of the nine infected members of staff, and the measures taken to stop the spreading of the disease and to ensure staff and patient safety.

## 2 METHODS

### 2.1 Detection and contact assessment

SARS‐CoV‐2 was detected by nasopharyngeal swab of a hematological patient whose clinical course is detailed in the results section. As soon as the disease was detected, all contact persons were identified and data about their SARS‐CoV‐2 positivity assessed by PCR and/or antibody testing in 92% of all category I contact persons. Those members of staff, who had contact with the patient but were off when COVID‐19 was diagnosed, were under orders to stay home for as long as was feasible and thus were not tested at the time. All contact persons, their symptoms as well as the time course of possible disease development were recorded.

### 2.2 PCR

RT‐PCR for the novel coronavirus was performed according to Corman et al.^10^

### 2.3 Antibody testing

IgG/IgM rapid tests were performed for the detection of antibodies.^11^ COVID‐19 IgG/IgM rapid test (Biomerica, Irvine, CA, USA) is a lateral flow chromatographic immunoassay to detect antibodies specific to SARS‐CoV‐2. We used serum samples throughout.

We have verified the rapid tests with a Roche antibody test using the Cobas e411 System (Test Elecsys Anti‐SARS‐CoV‐2, Kit # 09203095190, Roche, Penzberg, Germany).

### 2.4 Contact person definition

According to the German national health institute Robert‐Koch Institute (RKI)^12^ contact persons are persons who had contact to a confirmed case of COVID‐19 within 48 h before onset of symptoms in the index‐case. The end of the infectious period is currently not clear,^13^ especially in immunocompromised patients.

Contacts are defined as follows. Category I contacts with close contact (higher risk of infection): People with cumulative face‐to‐face contact for at least 15 min. These include members of the same household, persons with direct contact with secretions or body fluids, in particular with respiratory secretions from a confirmed COVID‐19 case, persons who are exposed to aerosol‐forming procedures, medical personnel with contact to a confirmed COVID‐19 case as part of care or a medical examination (≤2 m), and without the use of protective equipment.

Category II contacts (lower risk of infection). Persons who were in the same room as a confirmed COVID‐19 case, but had cumulative face‐to‐face contact with that case for under 15 min. Medical personnel who were in the same room as the confirmed COVID‐19 case without the use of adequate protective clothing, but who kept a distance of more than 2 m at all times.

Since there is an obligation to report COVID‐19, contact persons of category I were reported to the public health office, there was a close cooperation. The public health office, traced contact persons and also got in touch with the respective persons, thus had a structured overview of all individuals who had tested positive.

### 2.5 Hygiene regulations

Our LSCT‐Unit offers 16 beds for treatment according to our hygiene standards of care. These include that patients undergoing allogeneic stem cell transplantation are treated in the four HEPA filtered single rooms, but patients receiving an induction therapy for acute leukemias may be treated in double rooms, except when carrying infectious diseases. Prior to the COVID‐19 pandemic, our staff used basic personal protective equipment (PPE) that is, medical/surgical masks, apron‐style polyethylene gowns, non‐sterile gloves, and disinfectants when in contact with patients with proven transmissible infectious disease or when in contact with aplastic patients undergoing allogeneic stem cell transplantation. In cases of fever of unknown origin with no infectious agent detectable, no PPE was worn. Hence, when attending our AML patient, we did not wear PPE before COVID‐19 was detected, as to this time no transmissible infectious agents had been found.

During each shift, a patient is assigned one particular doctor and one nurse. However, due to shift changes, necessary diagnostic procedures, doctors’ visits, visits from the support team, and so on, our COVID‐19 patient had accumulated a considerable number of contacts. Despite this, work on the ward had to be continued. Therefore, detailed instructions were issued in close consultation with the local health authorities on who was quarantined and who was allowed to keep working. In addition to the usual safety measures on an LSCT‐Unit, as soon as the COVID‐19‐infection was detected, all patients on the ward were transferred to individual single rooms, no visitors were allowed, and patients were not allowed to leave the ward anymore. The usual equipment of non‐sterile gloves and disinfectants was used, as well as spunbond polyethylene gowns and FFP2 masks were made available for all staff members for every workday for the 2 weeks after detection of the COVID‐19 infection considered the high‐risk infectious time. Strict social distancing was decreed, a 2 m‐distance rule during breaks was strictly adhered to, contacts were reduced to a minimum, and employees were specifically trained for the situation. They were also not allowed to consume food while on the ward as that would have meant taking their masks off. Visitors to the hospital in general had already been prohibited. Staff who showed any kind of symptoms that could be typical for COVID‐19 at the time stayed home until cessation of symptoms for at least 2 days and the performance of a negative PCR test. As far as possible, contact persons were quarantined and replaced by other personnel. The remaining staff were allowed to continue working under the condition they adhere strictly to hygienic rules, wear masks at all times, and keep constant vigilance as to the development of symptoms.

As soon as antibody testing was possible, all available contacts of category I were tested except for one nurse who now lives in another city, one health‐care assistant who finished her contract, and the art therapist who has gone on a sabbatical (Table 1).

**TABLE 1:** Contacts and infections during the COVID‐19 outbreak at our clinic. Of the 36 category I contacts of our AML/COVID‐19 patient who had incubated the SARS‐CoV‐2 virus, and was admitted to our clinic, nine staff members were infected. Those contacts are listed in the Table and consisted of 10 medical doctors, 13 nurses, 2 health‐care assistants, 1 cleaning staff, 6 members of the diagnostic department, the transplant coordinator, and 3 members of the support team. The nine infected employees are shown in red, all of them were either positive in the PCR or in antibody tests (also in red). Almost all of them showed symptoms, the onset of symptoms is indicated in the symptoms column. We have used the same numbers in Figure 1 as in the table, to be consistent. In the column comments, we described some forms of contact to patient/staff, however more are described throughout the manuscript

#	Function in clinic	Age (y)	m/f	Date PCR	Result PCR	Symptoms	Ab‐test date/n.p.	Result Ab‐test	Comments
1	Doctor	34	f	15.03.	Neg	No	30.04.20	Neg	
2	Doctor	48	m	n.p.		No	09.04.20	Neg	Endoscopist who performed BAL
3	Doctor	35	f	n.p.		No	08.04.20	Neg	
4	Doctor	28	f	15.03.	Neg	Fever, cough, headache, fatigue, slight dyspnea, and onset of symptoms 22.03.			Quarantine for 14 days
				23.03	**Pos**				
				07.04.	Neg		09.04.20	**Pos**	
5	Doctor	44	f	15.03.	Neg	No			
				17.03.	Neg				
6	Doctor	49	m	15.03.	Neg	No			
				16.03	Neg				
							07.04.20	Neg	
				17.03.	Neg				
7	Doctor	32	m	n.p.		No	30.04.20	Neg	
8	Doctor	30	f	15.03.	Neg	Fever, cough, and onset of symptoms 17.03.	27.04.20	Neg	Quarantine until cessation of symptoms and PCR negativity
				25.03.	Neg				
9	Doctor	41	f	16.03.	Neg	No			
				17.03.	Neg		09.04.20	Neg	
10	Doctor	37	m	15.03.	Neg	No	09.04.20	Neg	Endoscopist who performed BAL
				17.03.	Neg				
				20.03.	Neg				
11	Nurse	26	f	16.03.	Neg	No	24.04.20	Neg	
12	Nurse	30	f	16.03.	Neg	No	24.04.20	Neg	
13	Nurse	27	f	16.03.	Neg	Slight sore throat and onset of symptoms 18.03.			Flat share with #16, quarantine for 14 days
							08.04.20	**Pos**	
14	Nurse	37	f	15.03.	Neg	Altered taste sensation, and onset of symptoms 22.03.			
							09.04.20	**Pos**	
15	Nurse	33	f	15.03.	Neg	No			
				16.03.	Neg				
							27.04.20	Neg	
				17.03.	Neg				
16	Nurse	29	f	16.03.	**Pos**	Fever, cough, sore throat, dyspnea, altered taste sensation, and onset of symptoms 16.03.			Quarantine for 14 days until cessation of symptoms and PCR negativity
				01.04.	Neg				
							09.04.20	**Pos**	
				02.04.	Neg				
17	Nurse	25	f	16.03.	Neg	No			
				17.03.	Neg		29.04.20	Neg	
18	Nurse	26	f	15.03.	Neg	Cough, sore throat, and onset of symptoms 16.03.			Quarantine until cessation of symptoms and PCR negativity
				20.03.	Neg		24.04.20	**Pos**	
19	Nurse	24	f	15.03.	Neg	No	07.04.20	Neg	
20	Nurse	37	f	17.03.	Neg	No	24.04.20	**Pos**	
21	Nurse	26	f	16.03.	Neg	No	07.04.20	Neg	
22	Nurse	41	f	16.03.	Neg	Fever, cough, and onset of symptoms 16.03.			Quarantine for 14 days until cessation of symptoms and PCR negativity
				17.03.	Neg				
							09.04.20	**Pos**	
				18.03.	Neg				
23	Nurse	24	n.p.				n.p.		
24	Diagnostic staff (EKG)	55	f	16.03.	Neg	Slight sore throat, fatigue, slight dyspnea, and onset of symptoms 20.03.			
	
							29.04.20	**Pos**	
25	Health‐care assistant	20	f	16.03.	Neg	No			
				17.03.	Neg		30.04.20	Neg	
26	Health‐care assistant	20	f	16.03.	Neg	No		Neg	
				17.03.	Neg				
							21.04.20		
27	Cleaner	46	f	18.03.	**Pos**	Fever, cough, chest pain, and onset symptoms 13.03.			Quarantine for 14 days until cessation of symptoms and PCR negativity
				02.04.	**Pos**				
							20.04.20	**Pos**	
				08.04.	Neg				
28	MTRA	35	f	n.p.		No	29.04.20	Neg	
29	MTRA	24	f	n.p.		No	30.04.20	Neg	
30	MTA Endoscopy	44	m	n.p.		No	04.05.20	Neg	
31	MTA Endoscopy	21	m	n.p.		No	29.04.20	Neg	
32	MTLA	58	f	15.03.	Neg	No	29.04.20	Neg	
33	Transplant coordinator	45	f	n.p.		No	21.04.20	Neg	
34	Art therapist	66	f	n.p.		No	n.p.		
35	Pastoral care	54	f	n.p.		No	29.04.20	Neg	
36	Psychologist	52	f	16.03.	Neg	No	27.04.20	Neg	Mild sore throat 12.03.
				19.03.	Neg				

## 3 RESULTS

### 3.1 Clinical course of the patient

**FIGURE 1:**
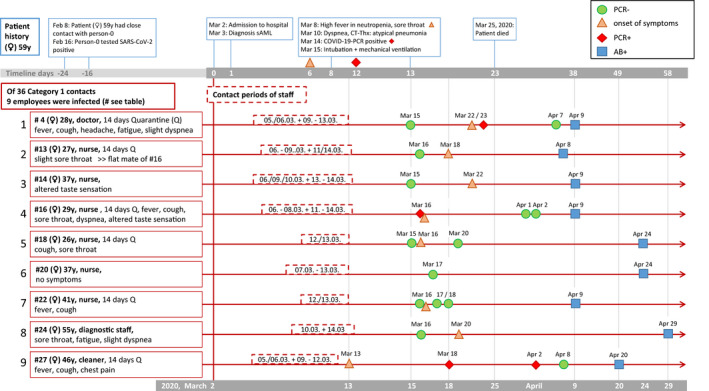
Timelines of infection. The upper part of the figure shows the clinical course of the patient 59y (♀) including admission to the hospital (day 0), diagnosis of acute myeloid leukemia (AML, day 1) time‐point of infection, symptoms and COVID‐19 infection (blue boxes). The patient showed first symptoms more than 3 weeks after her last contact to the proposed person‐0 who is the origin of infection. The SARS‐CoV‐2 PCR was positive day 12 after admission to the hospital. From the time‐point 2 days before the onset of symptoms until positive result of PCR the patient had close contact with 36 persons of the staff of the leukemia and stem cell transplantation unit and infected 9 persons of these 36 staff member. Eight of them had symptoms. The lower part of the figure (in red) shows the nine infected persons of the staff, the symptoms, the PCR, and antibody tests performed and the course of infection. PCR testing was not performed in all persons as staff member that were off duty and developed symptoms were advised not to go to the clinic and our mobile services came some weeks later. Importantly, due to our measures the outbreak resulted in no further infections of other patients despite ongoing clinical work

The patient was admitted to the clinic because of symptomatic anemia; further laboratory tests showed pancytopenia. Figure 1 shows the timelines of the clinical course, the COVID‐19 infection and infected staff members.

Bone marrow biopsy showed myelodysplastic changes as well as a 20%–30% infiltration by myeloid blasts (Figure S1 **)**. Abdominal ultrasound, chest X‐ray (Figure S2), pulmonary function testing, and echocardiography showed no abnormal findings, thus induction chemotherapy was started (Daunorubicin/Cytarabine 3 + 7). On day 3, the patient developed temperatures up to 39°C, at first without further symptoms apart from trouble swallowing and loss of appetite. Antibiotic treatment with Piperacillin/Tazobactam was initiated. Over the next 2 days oxygen saturation dropped repeatedly below 90% so extra oxygen was supplied via nasal cannula. The fever did not respond to antibiotic treatment, which was switched to Meropenem and Posaconazole was added. The patient continued to display high temperatures up to 39°C. A CT‐scan (Figure S2) showed pneumonia in the lower lobes, predominantly the left side and a bronchoalveolar lavage (BAL, Figure S3) was performed, in which neither any typical respiratory viruses nor bacteria were found. Over the following 2 days, the patient quickly deteriorated with oxygen saturation dropping further so that the patient was transferred to the intensive care unit. She developed a productive cough and still had a persistent high fever. At this point repeated questioning of the relatives revealed that the patient had contact to another person who had since tested positive for COVID‐19 (person‐0, Figure 1). As that contact had taken place more than 2 weeks before the patient's admission into hospital, the patient had not mentioned it. Our patient tested positive for SARS‐CoV‐2 and the following day needed to be intubated and started on mechanical ventilation. Over the next 10 days, the antibiotic regimen was repeatedly adjusted with no improvement of the respiratory situation. Very severe aplasia after chemotherapy persisted (Figure S3). Multi‐organ failure eventually set in and the patient died 10 days after diagnosis of COVID‐19.

### 3.2 All category I contacts among clinical staff from the LSCT‐Unit (Table)

During the time from initial hospital admission to diagnosis of COVID‐19, the patient had contact (category I) with 36 members of staff. Those contacts were **listed (Table)** and consisted of 10 medical doctors, 13 nurses, 2 health‐care assistants, 1 cleaning staff, 6 members of the diagnostic department, the transplant coordinator, and 3 members of the support team.

Contacts were listed only, if the contact had taken place within 48 h before the first symptoms of the patient or afterwards. Of these 36 contacts, 9 members of the staff of the LSCT‐Unit (1 MD, 6 nursing staff, 1 diagnostic, and 1 cleaning staff) were infected with COVID‐19, most of them symptomatic, and were tested positive by PCR and/or antibody testing. Due to changes in the management of coronavirus infection by local and central health offices and due to the temporary lack of laboratory capacities and ingredients, testing procedures are not the same for each staff member. Importantly, none of the other patients on the ward were tested positive or showed signs of COVID‐19 until end of June 2020.^13^

Four members of staff (Figure 1, #4, #16, #22, and #27) had severe symptoms but no one was hospitalized. The others had relatively mild symptoms. One younger person (#13, flat mate of #16) had almost no notable symptoms except for mild sore throat. One nurse (#20, 37y) had no symptoms at all, although she had cared for the patient for 1 week.

The symptoms were quite diverse; among them were fever, cough but also chest pain, and three staff members experienced dyspnea. Headache and altered taste sensation were also among the symptoms, some of which were only later brought into association with COVID‐19. 4/9 infected had cough, 4/9 sore throat, 3/9 fever, 3/9 dyspnea, 2/9 altered taste sensation, 2/9 fatigue, 1/9 chest pain, and 1/9 headache.^14^, ^15^

Based on our measures taken, none of the other patients on the ward were tested positive by PCR analysis in the further clinical course or showed signs of COVID‐19 until June 2020.

### 3.3 Cluster of outbreak on LSCT‐Unit and clinical history of patient

Figure 1 pictures the timeline of the outbreak including information about infected staff members but also the clinical course of the AML patient who succumbed to the disease. 8/9 staff members had clinical symptoms as described earlier. Not all staff members were tested by PCR, because some were at home on time off and not called back in just to be tested (mobile swab teams testing people in their homes were only installed later). However, 25 contact persons were tested, some of them repeatedly, 47 PCRs were performed in total, yielding positive results in 3 members of staff. 9/36 contact person tested positive for antibodies later on. All infected employees are described with relevant contact periods, tests, and symptoms in Figure 1. #2 was tested negative by PCR 10 days after the first of several contacts, then developed symptoms and was tested again on day 19 with a positive result. Interestingly, the nurse #6 developed typical symptoms, tested negative in PCR, and positive in antibody testing later.

In the process of the outbreak on our LSCT‐Unit, we had to alter or adjust test numbers and methods due to changes in regulatory requirements, testing capacities, material availability, and alterations in the recommendations for testing.

## 4 DISCUSSION

Stem cell transplantation units are especially sensitive care units for particularly vulnerable patients, therefore, in the current climate an understandable fear of a COVID‐19 outbreak is ever‐present. Our patient with newly detected AML incubated the SARS‐CoV‐2 for more than 2 weeks and experienced a late, unexpected and atypical outbreak, thereby exposing a comparatively large number of staff to the potential risk of infection.^16^

Due to the attentiveness and sensitivity of the team and the consistent implementation of hygiene measures, the outbreak resulted in no further infections of other patients despite ongoing clinical activity. All infected staff except one showed symptoms often described in COVID‐19 patients,^17^ their symptoms resolved and all personnel are back at work.

Of the 36 close contacts who were analyzed for SARS‐CoV‐2 positivity after outbreak discovery, 9 (25%) were tested positive by antibody testing and 8/9 of these staff members turned out to have been symptomatic, albeit some of them in such a mild way that it did not register at the time. PCR testing was not performed in all contact persons as staff members who were off duty or who could be replaced with other personnel were advised not to come to the clinic and stay in quarantine for 14 days. Staff members who became symptomatic while in the clinic were tested immediately and sent home to quarantine. The data collected showed a cluster of SARS‐CoV‐2 infections not all of which showed up in the PCR‐tests. Therefore, these data have to be discussed carefully. In one person, there were no detectable symptoms during the time of observation. In this person, it could be an asymptomatic infection but also false positive testing could be possible, which is a critical aspect if those tests are used as a “safety pass” for employees in the clinical setting in the future. However, in our test series, the antibody testing worked very well, similar to the literature^18^ but larger clinical studies are necessary.

Another point clearly demonstrated in our data are that the RT‐PCR of the nasopharyngeal swab on any given day is just a real‐time snapshot and has to be repeated frequently. For example, the MD who had contact to the COVID‐19 patient on several days over a period of more than 1 week, was infected, however was tested negative on day 10 after the first contact. She then developed symptoms and subsequently became positive in the SARS‐CoV‐2 RT‐PCR on day 19 after the first contact and was immediately quarantined. From this it can be concluded that testing for possible infection is essential, but diagnostic measures still need improvement, and a systematic evaluation of the PCR and antibody tests of both patients and employees is required.

Guidelines on testing and testing procedures have changed rapidly in recent weeks here, as in all countries. After the experiences we made with the management of COVID‐19 on our LSCT‐Unit, it has been very advantageous to introduce consistent testing at inpatient admission and to test patients immediately when symptomatic. This was not yet possible to the same extent at the beginning of the outbreak described here. Testing employees was and is particularly important, since our data show that young employees can have few symptoms and while being contagious. We suggest consistently performing PCR but also antibody tests for patients and also employees. Although testing strategies were frequently subject to change, a fairly coherent testing of all category I contacts and all patients on the LSCT‐Unit was eventually achieved.

Notable here is that with a combination of stringent hygienic measures and repeated testing no other patients were infected despite being cared for by the same personnel.

Our cluster of infection showed also interesting data regarding virus transmission. Different statements in current research literature about the transmission rate in families have been reported.^19^, ^20^ Of two nurses who both cared for the patient and who share a flat, only one became symptomatic quickly and tested positive. The other stayed in quarantine with her despite negativity in PCR. She developed the mildest of symptoms and had a positive antibody test later on. In contrast, some intensive contacts did not result in an infection. Surprisingly the employees who performed the bronchoalveolar lavage (BAL) on our AML/COVID‐19 patient remained free of symptoms and negative in all tests for SARS‐CoV‐2, although they had come into extremely close contact to the supposed infection site for a prolonged period of time.^21^, ^22^

Another important aspect highlighted in our data are that the clinical course of COVID‐19 in immunosuppressed patients could be different from that of other COVID‐19 cases. The estimated incubation period for COVID‐19 is supposedly no more than 14 days. However, looking back at the development of COVID‐19 in our AML patient and the onset of symptoms, the clinical findings suggest an incubation period of more than 3 weeks as there was a clear contact to a positive person and our patient had no other contacts to potential COVID‐19 sources after that. A possible explanation could be that in hematological patients a COVID‐19 infection might set off slowly, as the impaired immune system is unable to build up an immediate strong response. In acute leukemia, especially after allogeneic stem cell transplantation, immune reactions against immunogenic antigens like viral antigens but also other immunogenic antigenic structures, are weaker and differ in quality compared to those of healthy controls.^23^, ^24^, ^25^ Intensive mouthwashes, medication or other unknown factors could also explain a longer incubation period in leukemia patients. Ultimately, the underlying cause of death of our AML/COVID‐19 patient was pneumonia but also persistent aplasia which prevented her from building up a proper immune response; an interesting question here is whether COVID‐19 was a causative agent in prolonging aplasia. There exist no data about this so far. Therefore, further data from patients with different leukemias and other hematological malignancies but also from patients with other immune‐compromising diseases and conditions are necessary to elucidate this interesting issue.

In conclusion, outbreaks of COVID‐19 in hospital units with immunosuppressed patients, including hematological and transplant units, might become increasingly more common. In leukemia patients, these infections can possibly take a course different from that in immunocompetent healthy individuals. Due to our measures, no further infection among the patients has occurred. Consistent hygiene management and continuously improved testing in the future might help to save patients with severe hematological malignancies.

## 5 AUTHORSHIP CONTRIBUTION STATEMENT

Jochen Greiner designed the study, analyzed and interpreted the data, and wrote the manuscript. Marlies Götz analyzed the data, searched literature, and reviewed the manuscript. Waltraud Malner‐Wagner interpreted and collected the data. Constanze Wendt reviewed the manuscript. Martin Enders interpreted the data and reviewed the manuscript. Christine Durst interpreted and collected the data. Detlef Michel interpreted the data and reviewed the manuscript. Stephanie von Harsdorf interpreted the data and reviewed the manuscript. Susanne Jung designed the study and figures, analyzed and interpreted the data, and wrote the manuscript.

## CONFLICT OF INTEREST

The authors declare that there is no conflict of interest.

## ETHICAL APPROVAL

Ethical approval was sought for this study.

## Supporting information

Fig S1

Fig S2

Fig S3

## Data Availability

Not applicable.
