# Supplementary material for: Characteristics and mechanisms to control a COVID‐19 outbreak on a leukemia and stem cell transplantation unit
Source: Cancer Med. 2020 Dec 12;10(1):237–46. doi: 10.1002/cam4.3612 (PMC7826490; doi:10.1002/cam4.3612)
Supplement: Supplementary file 1 — Fig S1 [file CAM4-10-237-s001.pptx]

## Slide 1
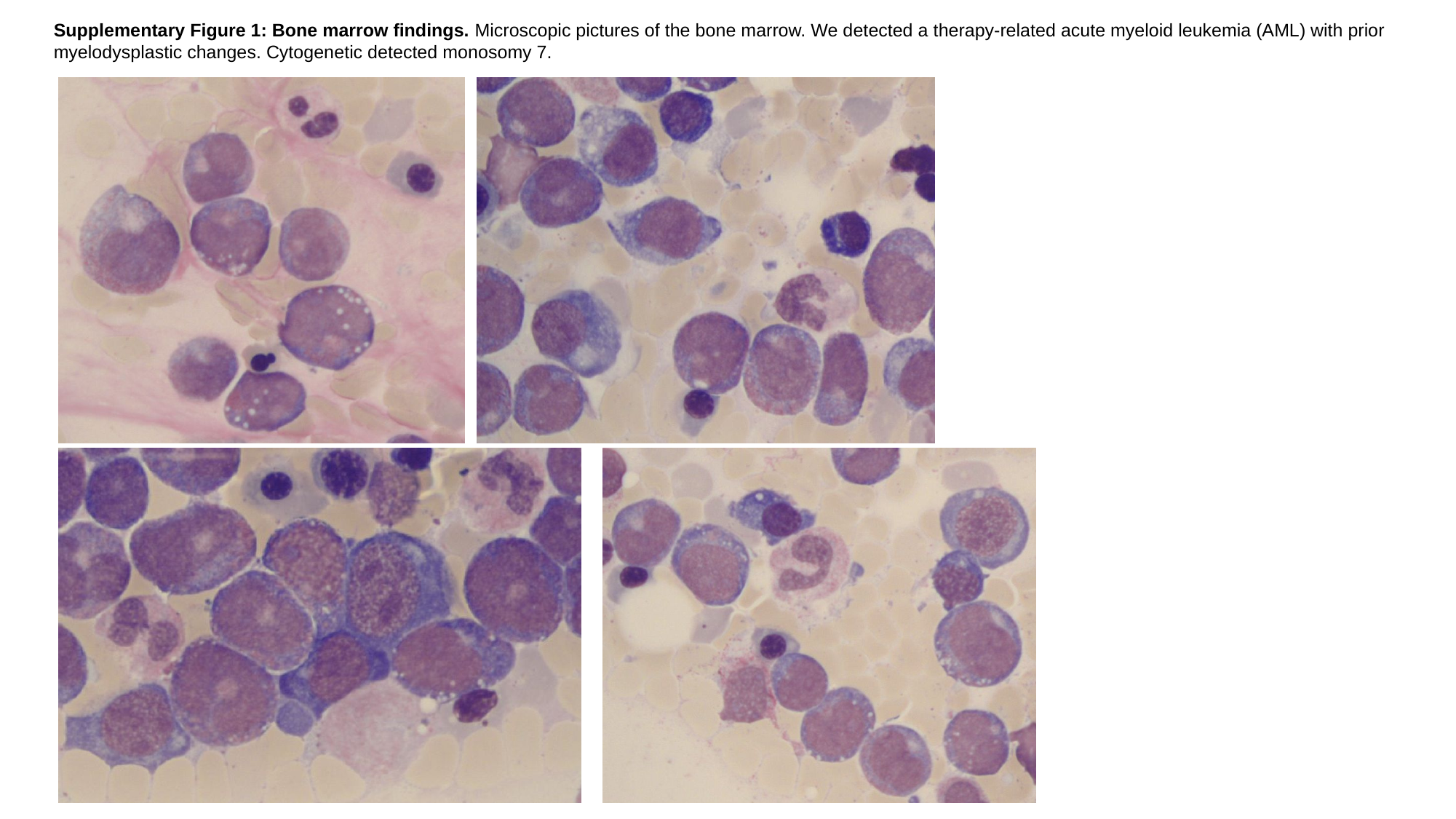

Supplementary Figure 1: Bone marrow findings. Microscopic pictures of the bone marrow. We detected a therapy-related acute myeloid leukemia (AML) with prior myelo­dys­plastic changes. Cytogenetic detected monosomy 7.
