# Supplementary material for: Characteristics and mechanisms to control a COVID‐19 outbreak on a leukemia and stem cell transplantation unit
Source: Cancer Med. 2020 Dec 12;10(1):237–46. doi: 10.1002/cam4.3612 (PMC7826490; doi:10.1002/cam4.3612)
Supplement: Supplementary file 2 — Fig S2 [file CAM4-10-237-s002.pptx]

## Slide 1
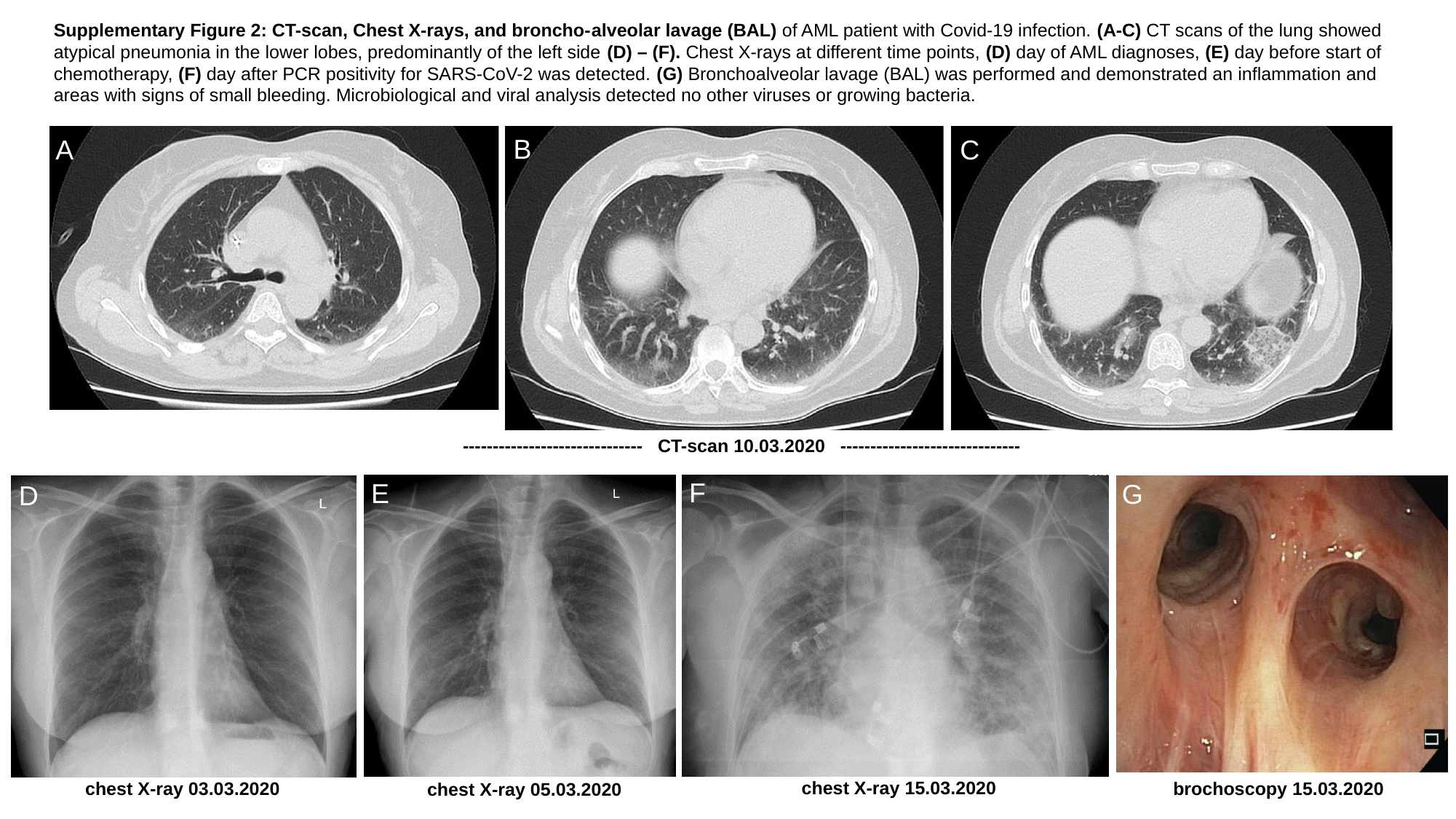

Supplementary Figure 2: CT-scan, Chest X-rays, and broncho-­alveolar lavage (BAL) of AML patient with Covid-19 infection. (A-C) CT scans of the lung showed atypical pneumonia in the lower lobes, predominantly of the left side (D) – (F). Chest X-rays at different time points, (D) day of AML diagnoses, (E) day before start of chemotherapy, (F) day after PCR positivity for SARS-CoV-2 was detected. (G) Broncho­alveolar lavage (BAL) was performed and demonstrated an inflammation and areas with signs of small bleeding. Microbiological and viral analysis detected no other viruses or growing bacteria.
B
C
A
 ------------------------------ CT-scan 10.03.2020 ------------------------------
F
E
G
D
chest X-ray 15.03.2020
chest X-ray 03.03.2020
brochoscopy 15.03.2020
chest X-ray 05.03.2020
