# Supplementary material for: Characteristics and mechanisms to control a COVID‐19 outbreak on a leukemia and stem cell transplantation unit
Source: Cancer Med. 2020 Dec 12;10(1):237–46. doi: 10.1002/cam4.3612 (PMC7826490; doi:10.1002/cam4.3612)
Supplement: Supplementary file 3 — Fig S3 [file CAM4-10-237-s003.pptx]

## Slide 1
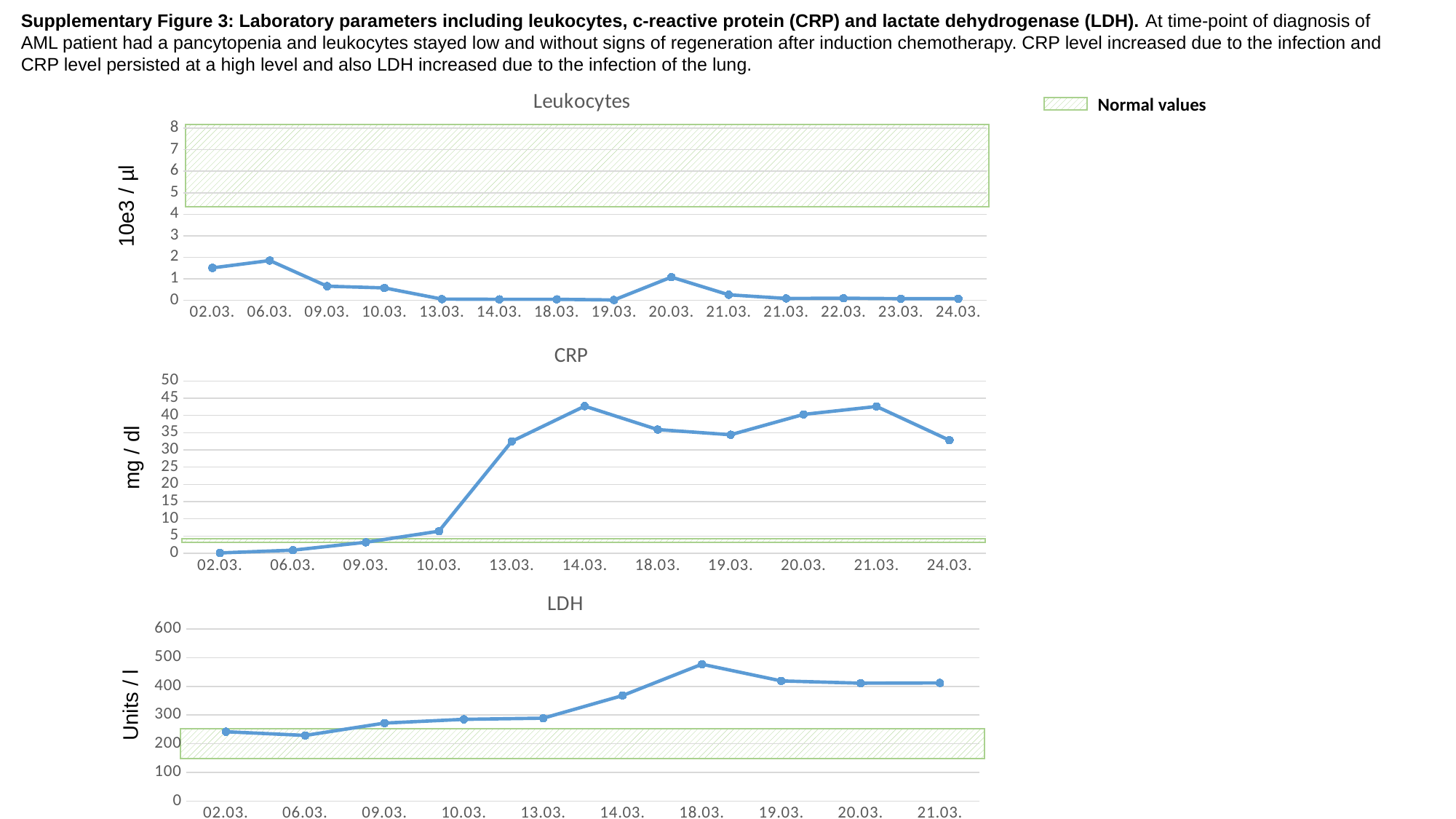

Supplementary Figure 3: Laboratory parameters including leukocytes, c-reactive protein (CRP) and lactate dehydrogenase (LDH). At time-point of diagnosis of AML patient had a pancytopenia and leukocytes stayed low and without signs of regeneration after induction chemotherapy. CRP level increased due to the infection and CRP level persisted at a high level and also LDH increased due to the infection of the lung.
### Chart: Leukocytes
| Category | Leukocytes 10e3/µl |
|---|---|
| 02.03. | 1.51 |
| 06.03. | 1.85 |
| 09.03. | 0.66 |
| 10.03. | 0.58 |
| 13.03. | 0.06 |
| 14.03. | 0.05 |
| 18.03. | 0.05 |
| 19.03. | 0.02 |
| 20.03. | 1.08 |
| 21.03. | 0.26 |
| 21.03. | 0.09 |
| 22.03. | 0.1 |
| 23.03. | 0.08 |
| 24.03. | 0.08 |
10e3 / µl
Normal values
### Chart:
| Category | CRP |
|---|---|
| 02.03. | 0.1 |
| 06.03. | 0.9 |
| 09.03. | 3.2 |
| 10.03. | 6.4 |
| 13.03. | 32.5 |
| 14.03. | 42.7 |
| 18.03. | 35.9 |
| 19.03. | 34.4 |
| 20.03. | 40.3 |
| 21.03. | 42.6 |
| 24.03. | 32.8 |mg / dl
### Chart:
| Category | LDH |
|---|---|
| 02.03. | 242.0 |
| 06.03. | 229.0 |
| 09.03. | 272.0 |
| 10.03. | 285.0 |
| 13.03. | 289.0 |
| 14.03. | 368.0 |
| 18.03. | 477.0 |
| 19.03. | 419.0 |
| 20.03. | 411.0 |
| 21.03. | 412.0 |Units / l
